# Supplementary material for: Selection of Salt-Tolerance and Ester-Producing Mutant Saccharomyces cerevisiae to Improve Flavour Formation of Soy Sauce during Co-Fermentation with Torulopsis globosa
Source: Foods. 2023 Sep 15;12(18):3449. doi: 10.3390/foods12183449 (PMC10529772; doi:10.3390/foods12183449)
Supplement: Supplementary file 1 [file foods-12-03449-s001.zip › foods-2609496-supplementary.pdf]

Selection of salt-tolerance and ester-producing mutant *Saccharomyces cerevisiae* to improve flavor formation of soy sauce during co-fermentation with *Torulopsis globosa*

Kun-Qiang Hong[1$*], Xiao-Meng Fu[1,2$], Fen-Fen Lei[1], Dong Chen[1], Dong-Ping He[1]

[1]Key Laboratory of Edible Oil Quality and Safety for State Market Regulation, Hubei Key Laboratory for Processing and Transformation of Agricultural Products, College of Food Science and Engineering, Wuhan Polytechnic University, 68 Xuefu South Road, Changqing Garden, Wuhan, 430023, P. R. CHINA

[2]School of Chemical Engineering and Technology, Tianjin University, Tianjin 300350, P.R. CHINA

* Corresponding author: Kun-Qiang Hong

E-mail: leo_hong92@163.com; Phone: +86-13237172182;

Address: Wuhan Polytechnic University, 68 Xuefu South Road, Changqing Garden, Wuhan, 430023, P. R. CHINA

[$]Kun-Qiang Hong and Xiao-Meng Fu contributed equally to the article.

Supplementary figures:

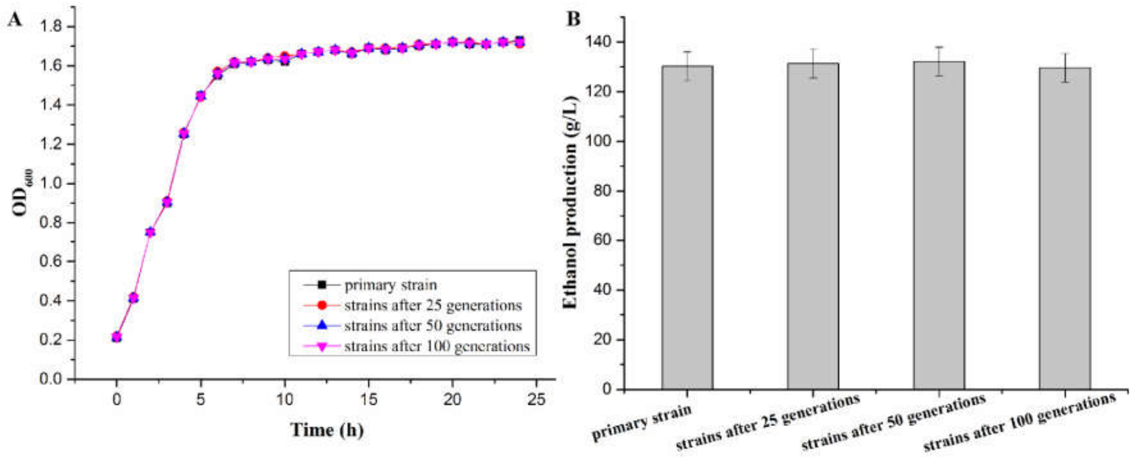

Figure S1 Stability analysis of strain HF-130. Growth curve (A), and ethanol production (B) of 1 (primary strains), 25 (strains after 25 generations), 50 (strains after 50 generations) and 100 (strains after 100 generations) strains were evaluated. All the data are the average values of three independent experiments.

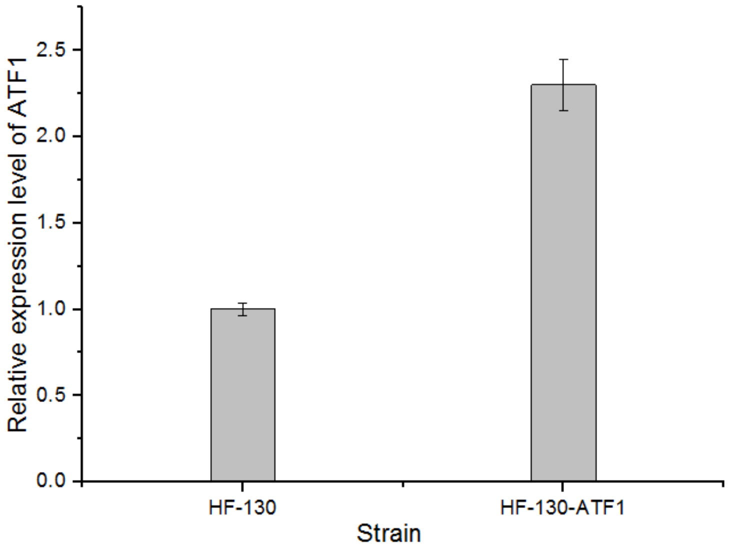

Figure S2 Relative expression level of *ATF1* gene in L-130 and L-130-ATF1. All the data are the average values of three independent experiments.

Table S1 RI and Odour description of flavour compounds

Flavour compounds	RI_{-EX}[a]	RI_{-LI}[b]	Odour description[c]
Ethanol	454	448	alcoholic
Ethyl acetate	598.8	605	fruity flavor
Isoamyl acetate	862	866	fruity flavor
Ethyl propionate	998.4	977	fruity flavor
Ethyl caprylate	1191.9	1193.8	fruity flavor
Phenylethyl acetate	1254.8	1270	aromatic taste
Ethyl caprate	1369	1400	waxy
Isobutyl acetate	1001	1013	fruity flavor
Dimethyl ether	481	478	ether taste, sweet taste
Trimethylsilyl decyl ester	1446	1450	odorless,or light scent in low concentration
Acetic acid	623	645	sour
Isoamyl alcohol	1151	1195	mild odor; alcoholic
phenylethyl alcohol	1095	1100	floral odor of roses
Phenylacetaldehyde	1045	1043	aromatic taste
2-Methylbutanol	698	700	ethereal
1-Octadecene	1793	1799	mild hydrocarbon odor
Octanoic acid	2072	2070	pungent odor, fruity aroma in low concentration

[a] Experimental value;

[b] Literature reported values in the NISTMS spectral database (https://webbook.nist.gov/chemistry/);

[c] Odour description refers to ChemicalBook (https://www.chemicalbook.com/)

Methods

S1 Preparation of repairing dsDNA (donors)

The oligonucleotide primers of *HXT7p, HXT7t, ATF1* were synthesized in the GENEWIZ company using standard desalting purification method, and diluted at 100 mM. Then 10 μL of 5 × Q5 reaction buffer, 10 μL of each of the two oligonucleotide primers, 10 μL of 10 mM dNTPs, 1 μL of Q5 enzyme, and 9 μL of ddH_2O were used for a 50 μL PCR reaction. The PCR reaction was set up by 98 °C for 10 s, 58 °C for 20 s, and 72 °C for 20 s, a total for 35 cycles. The PCR products of *HXT7p, HXT7t* and *ATF1* were purified by ethanol precipitation and diluted by ddH_2O at concentration 10 μg/μL.

S2 Construction of pCas9-XII5.5 plasmid

The XII-5.5 gRNA sequence of 5'TTGTCACAGTGTCACATCAG3' was selected from published paper. A high copy number 2 μ-based plasmid backbone with a mutated *Cas9* (D147Y and P411T) was were kindly provided by Ph. Yi-Jin Zhao from School of Life Sciences, Beijing University of Chemical Technology, China. The pCas9 vector was constructed with the *lacZα* sequence flanked by an *SNR52* promoter and a gRNA scaffold with *BsaI* cleavage site at the end of the *SNR52* promoter (GATC) and the beginning of the gRNA scaffold (GTTT)[27]. To assemble gRNAs on one

plasmid, the *lacZα* sequence was removed by *BsaI* digestion and replaced by PCR-generated fragments. The PCR templates were plasmids containing gRNA scaffold with a tRNA sequence or gRNA scaffold with a selection marker. The 20 bp gRNA targeting sequences were designed all on the primers and these two sites could be ligated on pCas9 vector to generate pCas9-XII5.5. The 4-bp sequences of other 20 bp gRNA targeting sequences can be used as Golden-Gate ligation sites to assemble different fragments. The sequences of oligonucleotide primers for generating donors and plasmids were listed in Table 2.

For a 20 μL total Golden gate reaction, 2 μL 10 × T4 Ligase buffer (M0202, New England Biolabs), 1.6 μL *BsaI* (R0535, New England Biolabs), 0.4 μL T4 Ligase (M0202, New England Biolabs), 150 ng for pCas9 plasmid (8.7 kb), and other fragments with a molar 1:1 ratio with the pCas9 were added into the reaction mix. The Golden gate reaction was carried out using the following temperature profile: Step 1, 37 °C for 30 min; Step 2, 37 °C for 10 min; Step 3, 16 °C for 5 min; Step 4, repeat steps 2 and 3 for 16 cycles; Step 5, 16 °C for 30 min; Step 6: 37 °C for 30 min, Step 7, 80 °C for 6 min; Step 8, 4 °C hold.

S3 2.3 VHG fermentation

100 g corn powder was mixed with 200 mL water at 65 °C, for 20 min. The mixture was incubated at 90 °C for 90 min with thermostable α-amylase. The temperature of the mixture was then lowered to 65 °C, and its pH was adjusted to 4.2–4.4 with 5 M H_2SO_4, and subsequently 0.36 g urea and saccharifying enzyme were added to the gelatinized starch. The medium was incubated at 65 °C for 30 min, and then cooled

down to 30 °C. Finally, a final NaCl concentration of 120 g/L was added for VHG fermentation. Extension of the incubation time to 9 h at 65 °C resulted in a complete release of glucose of 276 g/L in the medium.

Yeast strain was precultured in YPD medium for 24-48 h and then cells were centrifuged at 12,000 rpm for 5 min. The cells were washed with sterile water to remove trace glucose. The VHG fermentation was conducted in 500-mL flasks with 0.5 g/ L initial cell density and oxygen was limited through tightly sealing the flask. CO_2 was released by a 0.5 mm injection needle. Fermentation temperature was set at 30 °C and 40 °C, respectively. and samples were collected every 4 h and centrifuged at 12,000 rpm for 5 min. Extracellular glucose, glycerol, ethanol, and acetate concentrations were determined by a Waters Alliance 2695 high performance liquid chromatography (HPLC) (Waters, Milford, MA) using a Hitachi auto sampler, pump and refractive index detector and a Bio-Rad Aminex HPX-87H column (300 × 7.8 mm). The mobile phase was 5 mM H_2SO_4 at a flow rate of 0.4 mL/min. The column and detector temperatures were stabilized at 60 °C. The biomass was measured using method as described previously. The fermentation was processed till the weight loss of CO_2 was less than 1 g after interval 12 h.

S4 Sampling and analysis

HS-SPME-GC-MS (Headspace Solid Phase Microextraction Gas Chromatography-Mass Spectrometry) was used to extract flavor components from fermentation broth and analyze flavor components[32].

HS-SPME Sampling Conditions 8 mL of sample was put into a 20 mL vial and spiked with 3 g NaCl, and a small magnetic stirrer was added. The sample was equilibrated for 10 min and extracted for 50 min at 60 °C with continuous stirring. The SPME fiber holder equipped with DVB/CAR/PDMS fiber (Supelco, Inc., Bellefonte, PA, USA) was used for aroma compounds extraction in this study. After extraction, the fiber was inserted into the injection port of a GC-MS system (at 250 °C for 5 min).

Identification and quantitative analysis Identification of flavour compound was carried out using an Agilent 7890A GC coupled with an Agilent 5975C mass selective detector (MSD). The sample was analyzed on a CP-Wax column (50 m x 250 µm inner diameter, 0.2 µm film thickness). The injector temperature was 250 °C and the split mode was used (ratio 15:1). The oven temperature was held at 50 °C for 3 min, raised to 70 °C at a rate of 3 °C/ min increased to 170 °C at a rate of 3 °C/min, then increased to 240 °C at a rate of 8 °C/min and held at 240 °C for 3 min. The column carrier gas was helium at a constant flow rate of 1 mL/min. The mass spectrometer was operated in electron-impact mode at 70 eV. The temperatures of the interface, ion source and quadrupole were 280, 230 and 150 °C, respectively. The aroma compounds were determined by comparing the MS fragments detected with the mass spectra present in the NIST MS spectral database (https://webbook.nist.gov/chemistry/). The compounds identified by MS were further confirmed by comparing the retention times generated for each reference compound analyzed, using a commercial hydrocarbon mixture (C8–C40) for determination of the retention indices (RI). The quantitative analysis of the flavour compound was performed by calculation from the approximated curve using

the linear least-squares method. The respective quantitative values of the flavour compounds were determined by averaging the triplicate experiments.
